# Interval Type-2 Fuzzy PID Controller Using Disassembled Gradational Optimization

**DOI:** 10.3390/s23229067

**Published:** 2023-11-09

**Authors:** Yongzhi Chu, Hasiaoqier Han, Tianjiao Ma, Mingchao Zhu, Zhongcan Li, Zhenbang Xu, Qingwen Wu

**Affiliations:** 1 Changchun Institute of Optics, Fine Mechanics and Physics, Chinese Academy of Sciences, No. 3888, Dong Nanhu Road, Changchun 130033, China; chuyongzhi@ciomp.ac.cn (Y.C.); matianjiao@ciomp.ac.cn (T.M.); zhumingchao@ciomp.ac.cn (M.Z.); lizhongcan20@mails.ucas.ac.cn (Z.L.); xuzhenbang@ciomp.ac.cn (Z.X.); wuqw@ciomp.ac.cn (Q.W.); 2 Center of Materials Science and Optoelectronics Engineering, University of Chinese Academy of Sciences, Beijing 100049, China; 3 CAS Key Laboratory of On-Orbit Manufacturing and Integration for Space Optics System, Chinese Academy of Sciences, Changchun 130033, China

**Keywords:** disassembled gradational optimization method, interval type-2 fuzzy logic system, interval type-2 fuzzy PID controller, uncertain system, forced closed-loop system

## Abstract

This paper presents an interval type-2 fuzzy proportional–integral–derivative (IT2F-PID) controller that is designed using a new disassembled gradational optimization (D-GO) method. A PID controller is first optimized using the D-GO method and then connected to a type-1 fuzzy logic system (T1-FLS). The parameters of the T1-FLS are optimized, and the T1-FLS is blurred into the interval type-2 fuzzy logic system (IT2-FLS). Finally, the IT2F-PID controller is formed. The proposed method is compared with the concurrent and general optimization methods. The simulation results show that the D-GO method reduces the optimization time by over 90% compared with the general method, and decreases the integral-of-time-absolute-error (ITAE) by 30%. Beyond that, compared with the concurrent optimization method, the D-GO method reduces time by over 25%, and the ITAE value by about 95%. In the normal case, model uncertainty, target uncertainty, and external disturbance, the control ability of the IT2F-PID controller designed using the D-GO method is verified via simulations using a nonlinear forced closed-loop system. The results show that the overshoot is reduced by 80% and the fluctuation is reduced by 67% compared with a traditional PID controller and an IT2F-PID controller built using the general method.

## 1. Introduction

Conventional proportional–integral–derivative (PID) controllers have a simple structure, convenient adjustment, good stability, and reliable operation. Hence, they have been widely used in industrial control applications such as synchronous generator control, aircraft automatic navigation system control, computer numerical control, machine tool control, robot control, temperature control, and liquid level control [1,2,3,4,5]. Many intelligent controllers are based on conventional PID controllers. However, when the controlled object is a high-order complex system with a time delay, the performance of PID controllers is greatly reduced, and the control ability may even be lost [6,7].

To overcome this problem, many researchers have combined fuzzy logic systems (FLS) with conventional PID controllers [8,9]. Type-1 fuzzy PID (T1F-PID) and interval type-2 fuzzy PID (IT2F-PID) controllers have recently been developed [10,11]. The structure of the T1-FLS is similar to the IT2-FLS, with the IT2-FLS formed by adding uncertainties to the membership function of the T1-FLS [12,13]. Therefore, the IT2-FLS has a stronger control ability than that of the T1-FLS and can effectively control uncertain systems with inherent modeling nonlinearities and time delays [14,15]. The combination of the IT2-FLS and PID controllers is a current research hotspot [16]. For example, IT2F-PID controllers have been successfully applied to high-precision electro-optical tracking [17], a low-cost microcontroller [18], a highly complex nonlinear double-link robot operating system [19], and a mobile robot [20].

At present, the common structure of IT2F-PID controllers includes cascade and parallel types. The cascade-type IT2F-PID controllers connect the IT2-FLS with a proportional–integral controller in series and perform the proportional–integral operation on the output of the IT2-FLS [21,22,23]. This method does not control the three parameters of the PID controller separately when establishing the fuzzy control rules. So it does not take advantage of the independent control of the three parameters of the PID controller. The parallel IT2F-PID controller structure combines the IT2-FLS and the PID controller in parallel [6]. Compared with cascade-type controllers, the parallel-type IT2F-PID controller not only retains the benefits of PID controllers but also utilizes the powerful control ability of the IT2-FLS to adjust the control parameters of the PID controller in real time, thus enabling the PID controller to control high-order complex systems. Therefore, the parallel-type structure is more conducive to engineering applications. However, at present, the design of parallel IT2F-PID controllers employs an empirical method, and there is no other optimization approach [24].

By analyzing the optimal design of cascade-type IT2F-PID controllers, it appears that the construction of IT2F-PID controllers requires appropriate input and output factors and membership function parameters [23]. Therefore, the appropriate parameters must be selected for parallel-type IT2F-PID controllers. Several researchers have found that the input and output factors have significant effects on the performance of IT2F-PID controllers [25]. Therefore, on the basis of a fixed membership function, only the input and output factors need to be optimized, which is an efficient method of constructing the controller [26]. Parallel-type IT2F-PID controllers retain the structure of PID controllers, so there are eight parameters to be optimized, namely, the three coefficients of the PID controller and five scaling factors of the IT2-FLS. Cascade-type IT2F-PID controllers usually have only four parameters to be optimized; even so, it is very difficult to optimize four parameters at the same time using regular intelligent optimization algorithms [27]. Optimizing the eight parameters, therefore, presents a real difficulty in constructing a parallel-type IT2F-PID controller.

In this paper, a practical disassembled gradational optimization (D-GO) method is proposed for the design of a parallel-type IT2F-PID controller. The main feature of this method is the optimization of eight parameters in two steps. The first step is to optimize the proportional, integral, and differential coefficients of the PID controller. The second step then optimizes the scaling factor of error input, error change rate, proportional coefficient output, integral coefficient output, and differential coefficient output.

The merit of this method is that the optimal PID controller is determined in the first step, so the IT2-FLS only controls the increment of the proportional, integral, and differential coefficients. Compared with the general optimization method, which uses an intelligent optimization algorithm to optimize the parameters at the same time, the optimization range of the parameters is greatly reduced, which significantly reduces the optimization time. The D-GO method provides support for the IT2F-PID controller to realize online real-time parameter adjustment and control time-varying nonlinear complex systems. This paper compares the D-GO method with the concurrent and general optimization methods. Under the same conditions, the controller built using the D-GO method achieves better control performance and has a shorter optimization time. Through simulations, the validity of the parallel-type IT2F-PID controller designed using the D-GO method is verified using a forced closed-loop control system.

The balance of this paper is organized as follows. In Section 2, the IT2F-PID controller is introduced and the process of constructing a parallel IT2F-PID controller with the D-GO method is described in detail. Section 3 presents the forced closed-loop control system and the corresponding IT2F-PID controller constructed using the D-GO method. The simulation results are analyzed in Section 4, and the conclusions of this study are summarized in Section 5.

## 2. Interval Type-2 Fuzzy PID Controller

**Figure 1:**
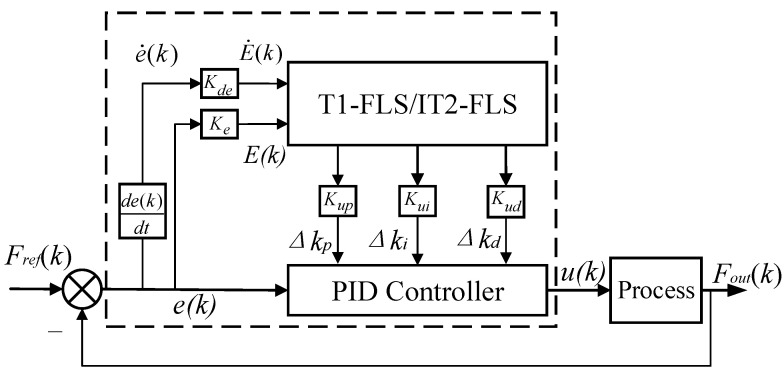
Interval type-2 fuzzy PID controller structure.

The parallel-type IT2F-PID controller is composed of an upper-level IT2-FLS controller and a lower-level classical PID controller. The upper intelligent controller provides a parameter change selection mechanism for the lower classical PID controller. The parameters of the lower classical PID controller are adjusted using the upper controller. The structure of the parallel IT2F-PID controller is shown in Figure 1.

The structure of the traditional PID controller is as follows [6]:

(1) $$\begin{array}{l} u(k)=k_{p}e(k)+k_{i}\int e(k)dk+k_{d}\frac{de(k)}{dk}\\ e(k)=Fref(k)-Fout(k) \end{array},$$

where *k_p_*, *k_i_*, and *k_d_* are the proportional, integral, and derivative gains, respectively, and *u(k)*, *Fref(k)*, *Fout(k)*, and *e(k)* are the controller output, expected target value, system output value, and the difference between the target value and the system output value, respectively. In the classic PID controller, the values of *k_p_*, *k_i_*, and *k_d_* are adjusted by technicians according to changes in the system. The control ability of the controller depends heavily on the experience of these technicians, which is not conducive to stable and accurate control [7]. Therefore, an intelligent IT2-FLS based on PID control rules is designed to adjust the parameters of the classical PID controller online, enabling better adaptation to changes in the system without excessive operator intervention and further enhancing the application range of the traditional controller. The composition and working process of the IT2-FLS is introduced below, and the proposed D-GO method is described in detail.

### 2.1. Interval Type-2 Fuzzy Logic System

**Figure 2:**
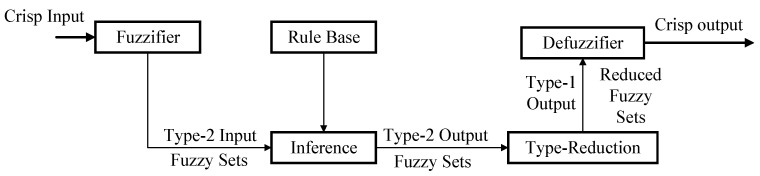
Structure of the type-2 fuzzy logic system.

The structure of the upper-level IT2-FLS consists of four parts: a fuzzifier, an inference engine, a rule base, and an output processor. The specific process is shown in Figure 2 [28].

The working process of the IT2-FLS is as follows [14]: the crisp input *e(k)* and *de(k)*/*dt* from the input sensor are first multiplied by the input factors (*K_e_* and *K_de_*) and fuzzified into the input interval type-2 fuzzy sets (IT2-FSs). The input IT2-FSs then activate the inference engine and rule base to generate the output IT2-FSs. The IT2-FSs rules are the same as in the T1-FLS, but the antecedents and/or consequents are represented by the IT2-FSs. The inference engine combines the fired rules and gives the mapping from the input IT2-FSs to the output IT2-FSs. The IT2 fuzzy outputs of the inference engine are processed using a type reducer, which combines the output sets and performs a centroid calculation to obtain the T1-FSs, called the type-reduction sets. After the type-reduction process, the type-reduction sets are defuzzified to obtain a clear output. The product of the crisp output and the scale factors (*K_up_*, *K_ui_*, and *K_ud_*) gives the adjustment values (Δ*k_p_*, Δ*k_i_*, and Δ*k_d_*) in the classic PID controller.

#### 2.1.1. Interval Type-2 Fuzzy Set

**Figure 3:**
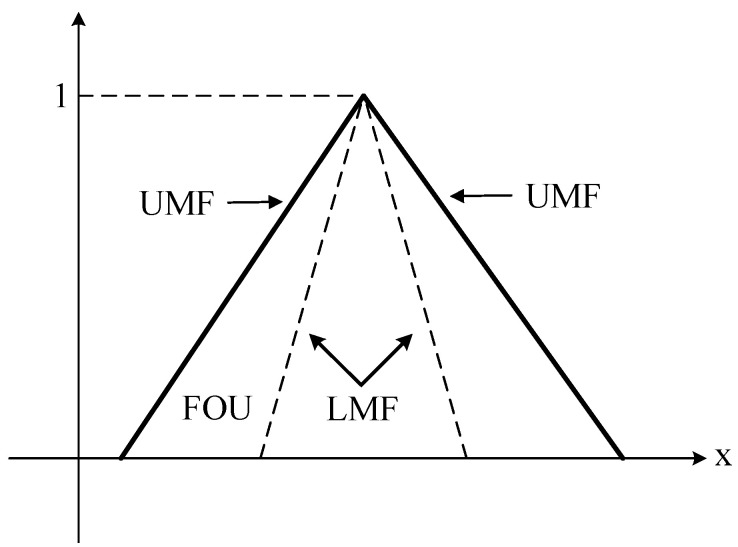
Interval type-2 fuzzy set.

An IT2-FS $\tilde{A}$ can be expressed as [29]:

(2) $$\tilde{A}=\int_{x\in X}\int_{u\in J_{x}}1/\left(x,u\right),$$

where *x* and *u* are the primary and secondary variables, respectively. Jx is the primary membership of *x*, *Jx* ⊆ [0, 1]. ∬ means taking all possible values of *x* and *u*. The secondary grades of $\tilde{A}$ are 1, fx(*u*) = 1(∀*u* ∈ *Jx* ⊆ [0, 1]). Figure 3 describes the footprint of uncertainty (FOU) of the fuzzy set $\tilde{A}$:

(3) $$FOU\left(\;\tilde{A}\right)=\left\{\left(x,u\right)|x\in X\;and\;u\in [{\underline{\mu }}_{\tilde{A}}\left(x\right),{\bar{\mu }}_{\tilde{A}}(x)]\right\},$$

where ${\underline{\mu }}_{\tilde{A}}(x)$ and ${\bar{\mu }}_{\tilde{A}}(x)$ are the lower membership function (LMF) and the upper membership function (UMF), respectively, and ${\underline{\mu }}_{\tilde{A}}(x)$ ≤ ${\bar{\mu }}_{\tilde{A}}(x)$. The LMF and UMF are type-1 membership functions. Like type-1 fuzzy sets, the interval type-2 membership function (IT2MF) can take different forms, such as triangular, trapezoidal, Gaussian, or bell. The membership function used in this paper is triangular, as shown in Figure 3.

#### 2.1.2. Rule Base

The rules of the IT2-FLS are generally IF-THEN rules that formulate the knowledge of technicians. The rules determine how the IT2-FLS adjusts the parameters of the PID controller. The parameters *k_p_*, *k_i_*, and *k_d_* mainly influence the system through the rise time, overshoot, and settling time. The influence of each parameter is described in Table 1.

The function of the *k_p_* is to speed up the response of the system. As *k_p_* increases, the response speed of the system becomes faster and the adjustment accuracy is enhanced. However, it is easy to overshoot the target value, potentially leading to system instability. If the value of *k_p_* is too small, the adjustment accuracy of the system will be reduced, the response speed will be slow, the adjustment time will be prolonged, and the static and dynamic characteristics of the system will deteriorate.

The function of *k_i_* is to eliminate the steady-state error of the system. With increasing *k_i_*, the static error of the system can be eliminated faster, but if the value is too large, the integral saturation phenomenon will occur at the initial stage of the response process, resulting in a large overshoot. If *k_i_* is too small, it will be difficult to eliminate the static error, thus affecting the adjustment accuracy of the system.

The function of *k_d_* is to improve the dynamic characteristics of the system. Its main function is to suppress changes in the deviation in any direction during the response process and predict the changes in deviation in advance. However, if *k_d_* is too large, the adjustment process will be overdamped, which will prolong the adjustment time and reduce the anti-interference performance of the system.

Based on the above PID parameter characteristics, the fuzzy control rules are formulated. The rule base takes the error and the change in error as the premise and the proportional, integral, and differential gains as the result. The structure of the fuzzy rules is as follows:

If *E*(*k*) is ${\tilde{A}}_{1}$ and $\dot{E}$(*k*) is ${\tilde{A}}_{2}$

then Δ*k_p_* is $\tilde{Y_{1}}$ and Δ*k_i_* is $\tilde{Y_{2}}$ and Δ*k_d_* is $\tilde{Y_{3}}$

where ${\tilde{A}}_{1}$, ${\tilde{A}}_{2}$, $\tilde{Y_{1}}$, $\tilde{Y_{2}}$, and $\tilde{Y_{3}}$ are IT2-FSs, as described in Figure 3.

#### 2.1.3. Fuzzification and Inference

When ${E(k)=x}_{1}^{\prime }$, the vertical line at $x_{1}^{\prime }$ crosses the $FOU({\tilde{A}}_{1})$ region to create an interval [${\underline{\mu }}_{{\tilde{A}}_{1}}(x_{1}^{\prime })$, ${\bar{\mu }}_{{\tilde{A}}_{1}}(x_{1}^{\prime })$]. When ${\dot{E}(k)=x}_{2}^{\prime }$, the vertical line at $x_{2}^{\prime }$ crosses the $FOU({\tilde{A}}_{2})$ region, creating an interval [${\underline{\mu }}_{{\tilde{A}}_{2}}(x_{2}^{\prime })$, ${\bar{\mu }}_{{\tilde{A}}_{2}}(x_{2}^{\prime })$]. After obtaining two sets of interval values, two firing levels are computed. The lower and upper firing levels are calculated as $\underline{f}(x^{\prime })=$ min[${\underline{\mu }}_{{\tilde{A}}_{1}}(x_{1}^{\prime })$, ${\underline{\mu }}_{{\tilde{A}}_{2}}(x_{2}^{\prime })$] and $\bar{f}(x^{\prime })=$ min[${\bar{\mu }}_{{\tilde{A}}_{1}}(x_{1}^{\prime })$, ${\bar{\mu }}_{{\tilde{A}}_{2}}(x_{2}^{\prime })$], respectively. After obtaining the upper and lower firing levels, the firing interval is obtained as $F(x^{\prime })=\left[\underline{f}(x^{\prime }),\bar{f}(x^{\prime })\right]$.

#### 2.1.4. Type Reduction and Defuzzification

The type-reduction process is part of the IT2-FLS that differs from the T1-FLS. The process of type reduction involves transforming IT2-FSs into T1-FSs [30]. The type reduction of IT2-FSs outputs T1-FSs, which are collections of all embedded T1-FSs. In the proposed controller, the most common center-of-set method based on the Karnik–Mendel (KM) algorithm is selected to map IT2-FSs to T1-FSs [31]. The calculation process is as follows:

(4) $$y_{l}=\underset{k\in \left[1,n-1\right]}{\min}\frac{\sum_{n=1}^{k}{\bar{f}}^{n}{\underline{y}}^{n}+\sum_{n=k+1}^{n}{\underline{f}}^{n}{\underline{y}}^{n}}{\sum_{n=1}^{k}{\bar{f}}^{n}+\sum_{n=k+1}^{n}{\underline{f}}^{n}},$$

(5) $$y_{r}=\underset{k\in \left[1,n-1\right]}{\max}\frac{\sum_{n=1}^{k}{\underline{f}}^{n}{\bar{y}}^{n}+\sum_{n=k+1}^{n}{\bar{f}}^{n}{\bar{y}}^{n}}{\sum_{n=1}^{k}{\underline{f}}^{n}+\sum_{n=k+1}^{n}{\bar{f}}^{n}},$$

(6) $$Y_{\cos}(x^{\prime })=\underset{\begin{array}{l} f^{n}\in F^{n}(x^{\prime })\\ y^{n}\in Y^{n} \end{array}}{\cup }\frac{\sum_{n=1}^{n}f^{n}y^{n}}{\sum_{n=1}^{n}f^{n}}=\left[y_{l},y_{r}\right],$$

where *Y_cos_(x)* is the T1-FS, *y_l_* and *y_r_* are the left and right endpoints of the IT2-FS, respectively. *y_l_* and *y_r_* can be calculated using the KM algorithm [32]. After the type-reduction process is applied, the clear output value of the IT2-FLS is calculated as:

(7) $$y(x)=\frac{y_{l}+y_{r}}{2},$$

### 2.2. Proposed D-GO Method

**Figure 4:**
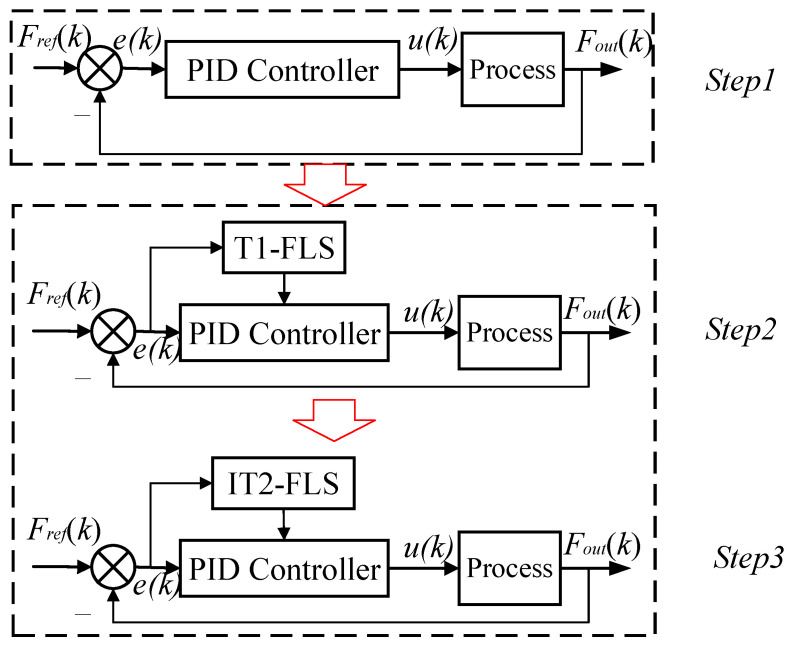
Diagram of the D-GO method.

Based on the structure of the IT2F-PID controller (Figure 1), at least eight parameters (*k_p_*, *k_i_*, *k_d_*, *K_e_*, *K_de_*, *K_up_*, *K_ui_*, and *K_ud_*) should be optimized to establish a suitable controller. To reduce the parameter optimization time and the hardware requirements, this paper disassembles the parameter adjustment into two categories and performs the optimization gradationally as shown in Figure 4.

The PID controller of the IT2F-PID controller plays the control role. The IT2-FLS adjusts the PID control parameters. Therefore, we optimize the PID controller parameters (*k_p_*, *k_i_*, and *k_d_*) first, and then optimize the IT2-FLS parameters. This effectively reduces the optimization time. The specific optimization process, optimization algorithm, and objective function are introduced below.

#### 2.2.1. Specific Process of the D-GO Method

The D-GO method proceeds as follows. First, the IT2F-PID controller is simplified to a PID controller, and the PID controller is connected to the controlled system as shown in Figure 4 (step 1).

At this time, only the PID controller parameters need to be optimized. After the PID parameters (*k_p_*, *k_i_*, and *k_d_*) have been optimized, the IT2-FLS can be established and optimized. Once the optimal PID controller parameters have been determined, the IT2-FLS can adjust Δ*k_p_*, Δ*k_i_*, and Δ*k_d_*. Compared with the cascade-type IT2F-PID controller parameters, the optimization range of these parameters is greatly reduced, which significantly reduces the optimization time.

The membership function of the IT2-FLS is uncertain. Hence, optimizing the input and output factors directly will take a lot of time, which affects work efficiency. The structure and mathematical foundations of the IT2-FLS are similar to those of the T1-FLS. Typically, the IT2-FLS is constructed by adding uncertainty to the membership function based on the T1-FLS [33,34,35]. The method of expanding a T1-FLS to an IT2-FLS is theoretically feasible and takes less time.

Therefore, we first establish a T1-FLS, select an appropriate membership function for the fuzzy sets E, $\dot{E}$, Up, Ui, and Ud, and then optimize the scale factors (*K_e_*, *K_de_*, *K_up_*, *K_ui_*, and *K_ud_*) of the T1-FLS as shown in Figure 4 (step 2). After that, the uncertainty of the membership functions in the T1-FLS is added, and then the IT2-FLS is established (step 3). To verify the effectiveness of the proposed D-GO method, *k_p_*, *k_i_*, *k_d_*, *K_e_*, *K_de_*, *K_up_*, *K_ui_*, and *K_ud_* are optimized under the condition that the T1-FLS and IT2-FLS membership functions have been determined. Various intelligent algorithms can be used to optimize the above parameters, including genetic algorithms [36], particle swarm optimization (PSO) algorithm [37], grey wolf optimization algorithm [38], and imperialist competition algorithm [39]. This paper uses the PSO algorithm as an example to demonstrate the implementation of the D-GO method and optimize the PID controller and IT2-FLS. The optimization objective function is the integral-of-time absolute error (ITAE).

#### 2.2.2. PSO and Objective Function

PSO is a global optimization algorithm inspired by the study of birds’ predation behavior [37]. The algorithm starts with randomly generated particles in the search space. In each iteration, particles move around the search space at the specified speed, hoping to find the optimal solution. The position of each particle is updated based on the particle’s current position, the particle’s individual optimal solution position, and the global optimal solution position. The particle’s position is then iteratively updated based on the new speed until the stop criterion is reached [37]. The mathematical expression of the above process is:

(8) $$\begin{array}{l} v_{i}^{t+1}=w\times v_{i}^{t}+c_{1}\times r_{1}\times (p_{i,d}-x_{i,d}^{t})+\\ c_{2}\times r_{2}\times (p_{g,d}-x_{i,d}^{t})\\ x_{i,d}^{t+1}=x_{i,d}^{t}+v_{i}^{t+1} \end{array},$$

where $x_{i,d}^{t}$ and $x_{i,d}^{t+1}$ are the current and future positions of the ith particle, respectively. The parameter $v_{i}^{t}$ is the current speed of the particle, and *w* is a weight function that controls the impact of the particle’s current speed on its future speed. The variable *p_i,d_* represents the best solution of particle *i* at iteration *t*, and *p_g,d_* is the best global solution attained so far. The weight factors c_1_ and c_2_ determine the importance of the current local optimal solution and the global optimal solution. Finally, *r*_1_ and *r*_2_ are random numbers in the range [0, 1], which further increase the randomness of the particle search in the whole search space.

The selection of a fitness function in the optimization algorithm directly affects the optimization effect; therefore, the commonly used ITAE [17] is selected as the fitness function. The ITAE is calculated as follows:

(9) $$ITAE=\int_{0}^{\infty }t\left|e(t)\right|dt,$$

## 3. Forced Closed-Loop System

To verify the effectiveness of the IT2F-PID controller established using the D-GO method proposed in this paper, a forced closed-loop system is established based on a single leg of a parallel manipulator. The control ability of the IT2F-PID controller established using the proposed method is tested when the controlled system and the input signal are uncertain. The controlled system and the IT2F-PID controller designed for the system are introduced in detail below.

### 3.1. System Model

The common leg structure of parallel manipulators incorporates a permanent magnet synchronous motor (PMSM) connected with a reducer and a ball screw [40]. To realize the forced control closed-loop system, a force sensor is installed at the end of the ball screw. The PMSM is the power output mechanism and is, therefore, the core of the whole system.

**Figure 5:**
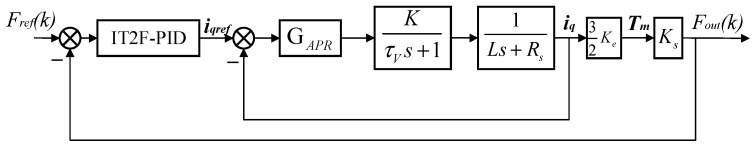
Block diagram of the forced closed-loop system.

The mathematical model of the motor is established and the PMSM is regarded as an ideal motor. It is assumed that the surface-mounted PMSM adopted does not consider the core reluctance, the loss of hysteresis and eddy current, the higher harmonics of the magnetic field, and the parameter changes in inductance and resistance. There is no damping winding in the rotor or the permanent magnet. The mutual inductance coefficient between the rotor winding and the stator winding is a sine (or cosine) function of the rotor position angle, and the friction coefficient is 0 [41]. The dynamic mathematical model of the PMSM is:

(10) $$\left\{\begin{array}{l} u_{d}=L_{d}\frac{d}{dt}i_{d}+R_{s}i_{d}-L_{q}p_{n}\omega_{e}i_{q}\\ u_{q}=L_{q}\frac{d}{dt}i_{q}+R_{s}i_{q}+L_{d}p_{n}\omega_{e}i_{d}+p_{n}\omega_{e}\psi_{f}\\ T_{e}=\frac{3}{2}p_{n}i_{q}\left[i_{d}(L_{d}-L_{q})+\psi_{f}\right]\\ T_{e}=T_{d}+J\frac{d\omega_{m}}{dt}+B\omega_{m} \end{array}\right.,$$

where *u_d_* and *u_q_* are the *d*-*q* axis components of the stator voltage, i_d_ and *i_q_* are the *d*-*q* axis components of the stator current, *Rs* is the stator resistance, *ω_e_* is the electrical angular velocity of rotor rotation, *ψf* is the flux chain, *L_d_* and *L_q_* are the *d*-*q* axis inductance components, and *T_d_*, *J*, *ω_m_*, *B*, and *Te* are the load torque, motor rotational inertia, motor rotor mechanical angular velocity, damping coefficient, and output torque, respectively. The whole forced closed-loop system model is established on the basis of the dynamic mathematical model of the PMSM [42]. Figure 5 shows a block diagram of the whole forced closed-loop system:

where G_APR_ is a PI controller, $\frac{K}{\tau_{V}s+1}$ is the simplified transfer function of the PWM inverter [43], *k* is the amplification ratio of the inverter, $\tau_{V}$ is the time constant, $\frac{1}{Ls+R_{s}}$ is the simplified transfer function of the PMSM, *L* is the armature inductance, *Rs* is the stator resistance, *Ke* is the torque coefficient, and *Ks* is the ratio coefficient of the ball screw and reducer.

Analysis of Equation (10) shows that the output torque is proportional to the current, so the inner loop of the forced closed-loop system is the current control loop. In this example, the force sensor signal is used for closed-loop force control, which is provided using the force (IT2F-PID) controller. Once the reducer and ball screw are connected to the motor, the motor output force is:

(11) $$F_{out}=T_{m}\times \frac{2\pi \cdot \eta \cdot i}{P_{h}},$$

where *T_m_* is the output torque of the motor, *P_h_* is the lead of the ball screw pair, *η* is the efficiency of the ball screw pair without preload, *i* is the reduction ratio of the reducer, and Fout is the outward output force of the ball screw pair. The transfer coefficient of the force sensor is 1.

### 3.2. Proposed Controller for the Forced Closed-Loop System

Figure 5 shows the forced closed-loop system with the IT2F-PID controller. The goal is for the output force of the structure to satisfy the design requirements. The steps involved in designing the IT2F-PID controller are as follows:

- First, the PID controller is designed. Then, the following steps are carried out: input the expected force, define the initial optimization range, optimize *k_p_*, *k_i_*, and *k_d_* using the PSO algorithm and ITAE of the fitness function, and obtain the optimal rounding for the PID parameters.
- Next, a Sugeno-type T1-FLS as the design basis of the IT2-FLS is established. The error and error change rate are selected as the input variables of the IT2-FLS, and the proportional, integral, and differential gains are selected as the output variables. After the input and output variables have been determined, a T1-FLS is established. Then, the linguistic variables are divided into quantitative levels. A finer quantization level will have more corresponding rules, and the control will become more complex. When the number of selected levels is small, there are fewer rules and the control effect will become rougher. Based on experience, we divided *E*, $\dot{E}$, *Up*, *U_i_*, and *U_d_* into three levels, namely, positive (P), zero (ZO), and negative (N). The relationships between the fuzzy linguistic values (*E*, $\dot{E}$, *Up*, *U_i_*, and *U_d_*) and the actual input and output values (e, $\dot{e}$, Δ*k_p_*, Δ*k_i_*, and Δ*k_d_*) are:
  (12) $$\left\{\begin{array}{c} E=K_{e}\cdot e\\ \dot{E}=K_{de}\cdot \dot{e}\\ ∆k_{p}=K_{up}\cdot U_{p}\\ ∆k_{i}=K_{ui}\cdot U_{i}\\ ∆k_{d}=K_{ud}\cdot U_{d} \end{array}\right.,$$

**Figure 6:**
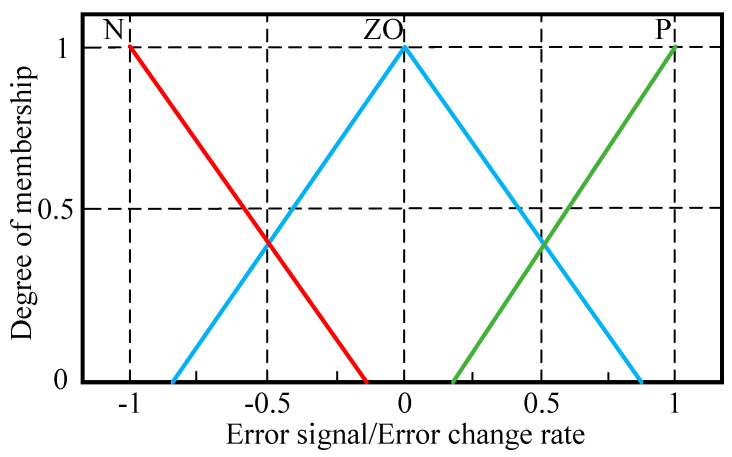
Membership functions for *E* and $\dot{E}$.

The membership functions used in the fuzzy sets of input variables *E* and $\dot{E}\;$are shown in Figure 6. The membership function is a trigonometric function with a range of [−1, 1]. The parameters of N are [−1, −1, −0.2], the parameters of ZO are [−0.8, 0, 0.8], and the parameters of P are [0.2, 1, 1].

**Figure 7:**
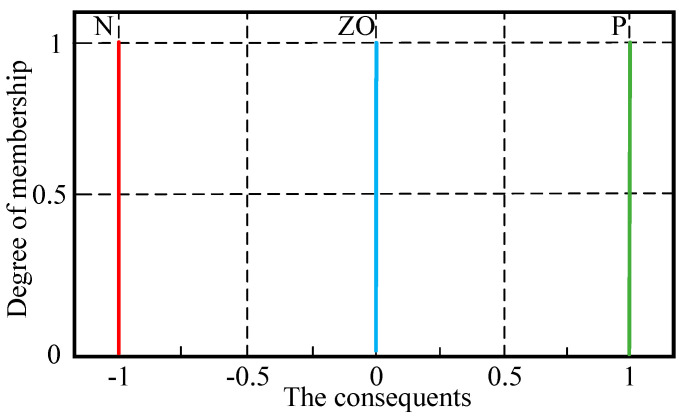
Membership functions for *U_p_*, *U_i_*_,_, and *U_d_*.

The membership functions used for the output (*Up*, *U_i_*, and *U_d_*) are shown in Figure 7.

The membership functions of the consequents are single values, and the corresponding values of $\left\{N,ZO,P\right\}$ are $\left\{-1,0,1\right\}$. The fuzzy rules are written based on Table 1 and experience. Based on the influence of the proportional, integral, and derivative gains on the force control system, the corresponding fuzzy rules are listed in Table 2, Table 3 and Table 4.

**Figure 8:**
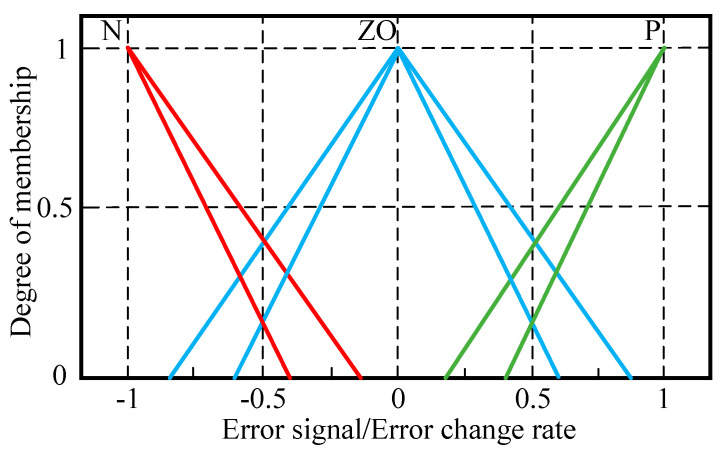
Membership functions of IT2-FLS for E and $\dot{E}$.

- After establishing the T1-FLS, the scale factors (*K_e_*, *K_de_*, *K_up_*, *K_ui_*, and *K_ud_*) of the T1-FLS are optimized. According to the PID parameters, the optimization ranges are defined for these five parameters, and the optimal values are obtained and rounded. Based on experience, the uncertainty of the membership functions in the T1-FLS is added, and then the IT2-FLS is established. The input membership function of the IT2-FLS is shown in Figure 8. At the bottom of the membership function of the T1-FLS, a blurring value of 0.2 is applied. The membership functions used for output (*U_p_*, *U_i_*, and *U_d_)* are shown in Figure 7.

## 4. Simulation Results

This section describes the application of the IT2F-PID controller established using the D-GO method to simulate and control a forced closed-loop system. At present, IT2F-PID controller construction mostly uses the general optimization method, which optimizes the parameters at the same time [27]. Beyond that, the concurrent method is added for comparison. Comparative analysis was conducted on the optimization time and ITAE of the D-GO and the concurrent and general optimization methods. Four different tasks were performed and the performance was compared with that of a PID controller and IT2F-PID controllers established using the general optimization method and the concurrent optimization method, respectively. To ensure the effectiveness of the comparison, the optimization algorithm and the objective function are the same for each controller. The parameters of the forced closed-loop system are listed in Table 5.

### 4.1. Task 1: Normal Case

**Figure 9:**
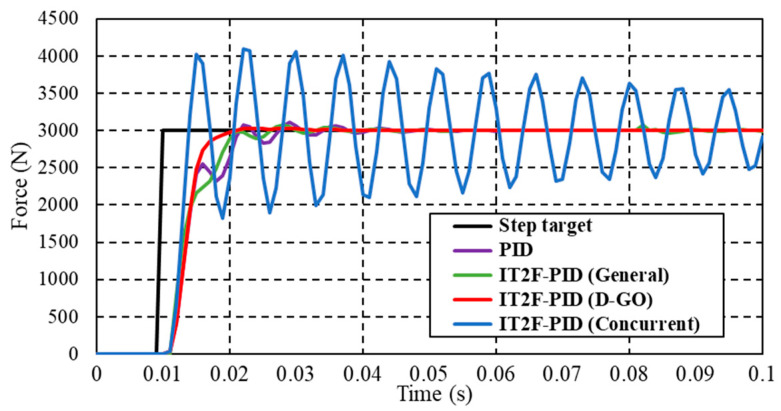
Response of the forced closed-loop system for a normal case.

First, we compare the performance of the PID controller and the IT2F-PID controllers established using the general optimization method, the D-GO method, and the concurrent optimization method, respectively, for a normal case with a 3000 N expected output force. The results are presented in Figure 9. The parameters (*k_p_*, *k_i_*, and *k_d_*) were optimized in the range [0–1, 0–500, 0–1] and the optimal PID parameters [0.04, 222, 0] were obtained after rounding. The scale factors (*K_e_*, *K_de_*, *K_up_*, *K_ui_*, and *K_ud_*) of the T1-FLS were then optimized. The initial optimization range of these five parameters was defined as [0–0.001, 0–0.000001, 0–0.1, 0–200, 0–0.01], and the optimal parameters were rounded. The IT2F-PID controller was then established as described in Section 3.2.

The general method is to set up the IT2F-PID controller directly and then optimize its eight parameters (*k_p_*, *k_i_*, *k_d_*, *K_e_*, *K_de_*, *K_up_*, *K_ui_*, and *K_ud_*) together. The general optimization method is different from the D-GO method. It does not optimize the PID controller first, so it has no preliminary understanding of the system error, error rate of change, or changes in the proportional, integral, and derivative gains. Therefore, the initial parameters (*k_p_*, *k_i_*, *k_d_*, *K_e_*, *K_de_*, *K_up_*, *K_ui_*, and *K_ud_*) are estimated values. The initial optimization range is [0–1, 0–500, 0–1, 0–1, 0–1, 0–1, 0–500, 0–1] and the optimal parameters are rounded.

The concurrent optimization is to connect the PID controller and IT2-FLS into the controlled system, respectively, for parameter optimization, and then combine them. The PID parameter optimization process is the same as that of the D-GO method, so it is not described here. The IT2-FLS parameter optimization process is similar to that of the general method, which is also not described here. The parameters to be optimized are *K_e_*, *K_de_*, *K_up_*, *K_ui_*, and *K_ud_* and the initial optimization range is [0–1, 0–1, 0–1, 0–500, 0–1]. The IT2F-PID controller built using the D-GO method has obvious advantages over the controllers built using the concurrent and general optimization methods, as shown in Figure 9. Compared with the other controllers, the controller reduces the overshoot by 80% and the fluctuation by 67%. It has only a small overshoot, no oscillation, and no steady-state error after stabilization. Compared with the IT2F-PID controller, the PID controller has a longer rise time, larger overshoot, and longer settling time. The parameters of the PID controller used are consistent with those of the IT2F-PID controller built using the D-GO method; therefore, the reason for the poor performance is that the PID controller is not connected with the IT2-FLS. Similarly, the IT2F-PID controller established using the general method also has problems like the PID controller and it exhibits sudden fluctuations even after the system has stabilized. Although the fluctuations quickly disappear, they have a certain impact on the stability of the system. The reason for the above problems is that compared with the D-GO method, the initial parameter selection is not accurate enough, resulting in the optimized parameters being not optimal and the control performance being poor. For the D-GO method, a better parameter range was preliminarily determined in the PID parameter optimization step. The controller constructed using the concurrent method does not converge. The control result of the IT2F-PID controller is poor because the PID controller and IT2-FLS are connected to the controlled system, respectively, for parameter optimization during the construction process. The combination of the two causes the IT2F-PID controller to lose control of the controlled system. This illustrates that the proposed controller achieves superior performance.

To illustrate the advantages of the D-GO method, it is compared with the concurrent and general optimization methods in Table 6. Taking the normal case as an example, the D-GO method has a significantly shorter optimization time than the concurrent and general optimization methods in constructing the IT2F-PID controller and outperforms the concurrent and general optimization methods in terms of the ITAE. The D-GO method reduces the optimization time by over 90% and the ITAE value by about 30% compared with the general method. Compared with the concurrent optimization method, the D-GO method reduces time by over 25% and the ITAE value by about 95%.

By comparing the optimization time of the three methods, it can be found that when the IT2F-PID controller is disassembled into the PID controller and IT2-FLS, fewer parameters are optimized at the same time, and a lot of optimization time can be saved. By comparing ITAE values, it can be found that if the relationship between the PID controller and IT2-FLS is not considered and the controller is only optimized and recombined, the controller will lose control ability. The optimization method that saves time while ensuring control ability is the D-GO method, which first disassembles the controller and analyzes the relationship between the PID controller and the IT2-FLS, and then carries out gradational optimization. It ensures that fewer parameters are optimized at the same time, and the relationship between the two controllers is considered to ensure the control performance.

### 4.2. Task 2: Model Uncertainty

In practical control applications, the existence of uncertainties such as temperature and friction [44] makes it difficult to obtain accurate values from the physical model of the forced closed-loop system. Therefore, there is a mismatch between the simulation results and the actual model output. To verify the effectiveness of the D-GO method, the motor inductance parameter and the winding resistance were set to 1.1 times their nominal values. All control parameters remained unchanged.

**Figure 10:**
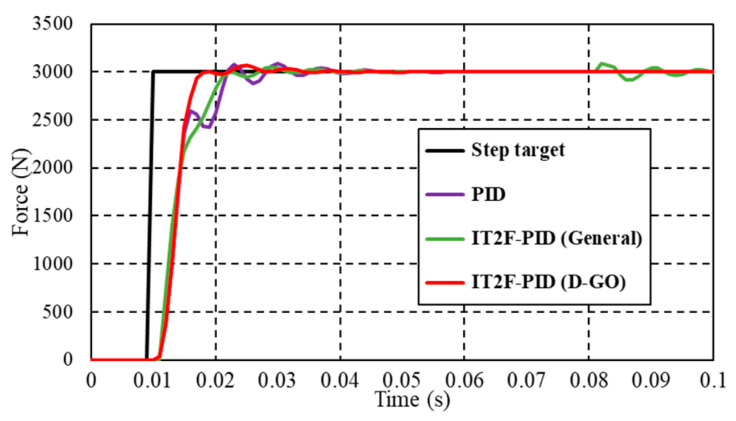
Force tracking curve under model mismatch.

The results in Figure 10 show that, in the case of model mismatch, the force tracking performance of the controller of the D-GO method is consistent with that of model matching. The force response curve has only a small overshoot, and there is no steady-state error after a slight oscillation, so the required value is quickly attained and accurately tracked. The PID controller and the IT2F-PID controller established using the general optimization method exhibit worse force tracking ability than the IT2F-PID controller established using the D-GO method. In addition, the IT2F-PID controller established using the general method still exhibits sudden fluctuations and it is more obvious after the system has stabilized. Although the fluctuations quickly disappear, they have a certain impact on the stability of the system. The reasons for the above phenomena are the same as that in normal cases. The PID controller is not connected with the IT2-FLS, so it does not have real-time tuning ability, and its control performance is inevitably poor. The parameters of the IT2F-PID controller established using the general method are not optimal, which leads to poor control performance when the controlled system parameters are uncertain, and the fluctuation after stability is more obvious. In normal cases, the concurrent method has not converged, and it still does not converge in the model uncertainty; therefore, it is not discussed here. This again illustrates that the proposed controller achieves superior performance.

### 4.3. Task 3: Target Uncertainty

In the actual force control process, there is great uncertainty about the expected output force. Thus, we performed experiments to verify the adaptability of the D-GO method to different expected forces. The expected input was a pulse force with an amplitude of 3000 N, a period of 0.1 s, and a pulse width of 50%. This input is significantly different from that used to establish the controller and is intended to reflect the effectiveness of the proposed method.

**Figure 11:**
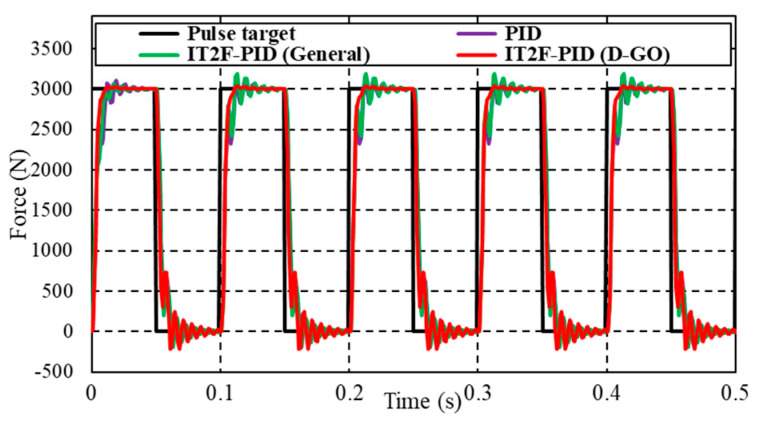
Force tracking curve under pulse signal.

The results in Figure 11 show that the IT2F-PID controller designed using the D-GO method achieves excellent force tracking performance under the peak of pulse force. The force tracking fluctuates at the bottom, so it can be seen that the optimization parameters obtained in the normal case cannot be applied to any situation. Compared with the other controllers, the proposed IT2F-PID controller reduces the overshoot by 80% and the fluctuation by 67%. The force response curve has a small overshoot, and quickly tracks the required value thereafter. The performance is obviously better than that of the PID controller and the IT2F-PID controller designed using the general optimization method. Because the PID controller does not have the ability to adjust its own parameters, the parameters of the IT2F-PID controller designed using the general method are far from the optimal value, resulting in an obvious overshoot and a significant increase in the stability time of both.

### 4.4. Task 4: External Disturbance

The forced closed-loop control system is inevitably subjected to external force interference during operation. Under external disturbance, the validity of the D-GO method is tested. The increase in disturbance force was F_d_ = 300 N in the normal case. The parameters of each controller remain unchanged.

**Figure 12:**
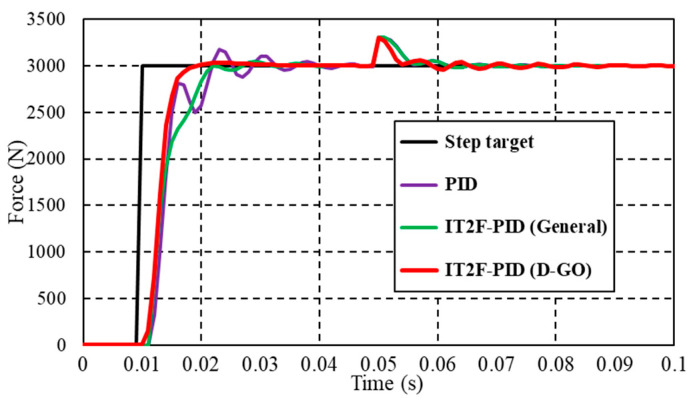
Force tracking curve under external disturbance.

The force tracking performance is shown in Figure 12. Under external disturbance, the controller constructed using the D-GO method has a short rise time, small overshoot, and short settling time in the process of force tracking, and the overall tracking performance is obviously better. Compared with the PID controller and the controller constructed using the general method, the overshoot was reduced by 80%, the fluctuation was reduced by 67%, and especially the recovery speed after interference was obviously better. The reason for the poor performance of the controller established using the general method is similar to the normal case. The PID has no ability to adjust its own parameters, and the controller parameters established using the general method have a wide range of optimization, which leads to a long distance from the optimal value and poor control performance. The parameters of the controller constructed using the D-GO method are closer to the optimal value and the control performance is better.

## 5. Conclusions

This paper has described the D-GO method for designing a parallel-type IT2F-PID controller. The proposed method disassembles the parallel IT2F-PID controller into a PID controller and a T1-FLS for parameter optimization and then blurs the membership function of the T1-FLS to construct a parallel IT2F-PID controller. Compared with the general optimization method, the D-GO method reduces the optimization time by over 90% and the ITAE value by about 30%. Compared with the concurrent optimization method, the D-GO method reduces time by over 25% and the ITAE value by about 95%. The D-GO method significantly reduces the optimization time and supports the IT2F-PID controller to realize online real-time parameter adjustment and control a time-varying nonlinear complex system.

Simulations were conducted to verify the control performance of the D-GO method. Through comparisons with a PID controller and an IT2F-PID controller established using the concurrent and general optimization methods, we found that in the normal case, model uncertainty, target uncertainty, and external disturbance, the D-GO method’s controller achieves superior control performance for the forced closed-loop system. The overshoot is reduced by 80% and the fluctuation is reduced by 67% compared with the PID controller and the parallel IT2F-PID controller established using the concurrent and general optimization methods. In addition, compared with the other controllers, the D-GO method’s controller does not produce sudden fluctuations after the system has been stable and the recovery speed after interference is obviously better. This illustrates that the D-GO method’s controller achieves superior performance. The results presented in this paper are sufficient to demonstrate that the D-GO method is suitable for the establishment of IT2F-PID controllers. By extension, it also provides inspiration for the establishment of other intelligent controllers.

## Figures and Tables

**Table 1:** Effects of gain parameters.

Gain Parameters	Effects of Increasing Gain		
	Rise Time	Overshoot	Settling Time
$k_{p}$	Decrease	Increase	Small change
$k_{i}$	Decrease	Increase	Increase
$k_{d}$	Decrease	Decrease	Decrease

**Table 2:** Rule base for the proportional gain.

Error Signal	Change in Error Signal		
	N	ZO	P
N	N	N	N
ZO	N	P	P
P	P	P	P

**Table 3:** Rule base for the integral gain.

Error Signal	Change in Error Signal		
	N	ZO	P
N	ZO	ZO	ZO
ZO	P	P	P
P	ZO	ZO	ZO

**Table 4:** Rule base for the derivative gain.

Error Signal	Change in Error Signal		
	N	ZO	P
N	N	N	P
ZO	N	ZO	P
P	N	N	P

**Table 5:** Parameters of the forced closed-loop control system.

Parameter	Meaning	Value	Unit
*L*	Rotator inductance	0.01225	H
*R*	Rotator resistance	12.4	Ω
*Pn*	Number of pairs	5	/
*Ke*	Torque constant	0.1125	/
*τ*_*V*_	Inverter time constant	0.001	s
*K*	Inverter gain	1	/
*Ks*	Proportionality coefficient	113,100	/
*G*_*APRP*_	The proportional parameter of *G*_*APR*_	39	/
*G*_*APRI*_	The integral parameter of *G*_*APR*_	8563	/

**Table 6:** Comparison between the D-GO method and the concurrent and general optimization methods.

Method	Time (s)	ITAE
The D-GO	1020	0.1462
The concurrent optimization	1370	2.8541
The general optimization	13,242	0.2087

## Data Availability

The authors confirm that the data supporting the findings of this study are available within the article.

## References

[1] Skogestad S. (2003). Simple analytic rules for model reduction and PID controller tuning. J. Process Control.

[2] Toscano R. (2007). Robust synthesis of a PID controller by uncertain multimodel approach. Inf. Sci..

[3] Reznik L., Ghanayem O., Bourmistrov A. (2000). PID plus fuzzy controller structures as a design base for industrial applications. Eng. Appl. Artif. Intell..

[4] Xie T.C., Nan X. (2009). Research and Applications of Fuzzy-PID Control on NC Machine. Key Eng. Mater..

[5] Yamamoto T., Kawada K., Kugemoto H., Kutsuwa Y. (2009). Design and Industrial Applications of a Control Performance Assessment Based PID Controller. IFAC Proc. Vol..

[6] El-Bardini M., El-Nagar A.M. (2014). Interval type-2 fuzzy PID controller for uncertain nonlinear inverted pendulum system. ISA Trans..

[7] Kim J.H., Oh S.J. (2000). A fuzzy PID controller for nonlinear and uncertain systems. Soft Comput..

[8] Patel A.V., Mohan B.M. (2002). Analytical structures and analysis of the simplest fuzzy PI controllers. Automatica.

[9] Mohan B.M., Patel A.V. (2002). Analytical structures and analysis of the simplest fuzzy PD controllers. IEEE Trans. Syst. Man Cybern. Part B.

[10] Güzelkaya M., Eksin I., Yeşil E. (2003). Self-tuning of PID-type fuzzy logic controller coefficients via relative rate observer. Eng. Appl. Artif. Intell..

[11] Kumbasar T. (2014). A simple design method for interval type-2 fuzzy PID controllers. Soft Comput..

[12] Linda O., Manic M. (2011). Uncertainty-robust design of interval type-2 fuzzy logic controller for delta parallel robot. IEEE Trans. Ind. Inform..

[13] Lu X.G., Liu M., Liu J.X. (2017). Design and optimization of interval type-2 fuzzy logic controller for delta parallel robot trajectory control. Int. J. Fuzzy Syst..

[14] Jammeh E.A., Fleury M., Wagner C., Hagras H., Ghanbari M. (2009). Interval type-2 fuzzy logic congestion control for video streaming across IP networks. IEEE Trans. Fuzzy Syst..

[15] El-Nagar A.M., El-Bardini M., El-Rabaie N.M. (2014). Intelligent control for nonlinear inverted pendulum based on interval type-2 fuzzy PD controller. Alex. Eng. J..

[16] Hagras H.A. (2004). A hierarchical type-2 fuzzy logic control architecture for autonomous mobile robots. IEEE Trans. Fuzzy Syst..

[17] Tong W., Zhao T., Duan Q., Zhang H., Mao Y. (2022). Non-singleton interval type-2 fuzzy PID control for high precision electro-optical tracking system. ISA Trans..

[18] El-Nagar A.M., El-Bardini M. (2014). Practical implementation for the interval type-2 fuzzy PID controller using a low cost microcontroller. Ain Shams Eng. J..

[19] Kumar A., Kumar V. (2017). A novel interval type-2 fractional order fuzzy PID controller: Design, performance evaluation, and its optimal time domain tuning. ISA Trans..

[20] Kumbasar T., Hagras H. (2014). Big Bang–Big Crunch optimization based interval type-2 fuzzy PID cascade controller design strategy. Inf. Sci..

[21] Khooban M.H., Alfi A., Abadi D.N.M. (2013). Teaching–learning-based optimal interval type-2 fuzzy PID controller design: A nonholonomic wheeled mobile robots. Robotica.

[22] AbouOmar M.S., Su Y., Zhang H., Shi B., Wan L. (2022). Observer-based interval type-2 fuzzy PID controller for PEMFC air feeding system using novel hybrid neural network algorithm-differential evolution optimizer. Alex. Eng. J..

[23] El-Nagar A.M., El-Bardini M. (2014). Derivation and stability analysis of the analytical structures of the interval type-2 fuzzy PID controller. Appl. Soft Comput..

[24] Araujo H., Xiao B., Liu C., Zhao Y. (2014). and Lam, H.K. Design of type-1 and interval type-2 fuzzy PID control for anesthesia using genetic algorithms. J. Intell. Learn. Syst. Appl..

[25] Yesil E. (2014). Interval type-2 fuzzy PID load frequency controller using Big Bang–Big Crunch optimization. Appl. Soft Comput..

[26] Kumar A., Kumar V. (2017). Evolving an interval type-2 fuzzy PID controller for the redundant robotic manipulator. Expert Syst. Appl..

[27] Hamza M.F., Yap H.J., Choudhury I.A. (2017). Cuckoo search algorithm based design of interval Type-2 Fuzzy PID Controller for Furuta pendulum system. Eng. Appl. Artif. Intell..

[28] Mendel J.M. (2007). Type-2 fuzzy sets and systems: An overview. IEEE Comput. Intell. Mag..

[29] Mendel J.M., John R.I.B. (2002). Type-2 fuzzy sets made simple. IEEE Trans. Fuzzy Syst..

[30] Wu H., Mendel J.M. (2002). Uncertainty bounds and their use in the design of interval type-2 fuzzy logic systems. IEEE Trans. Fuzzy Syst..

[31] Mendel J.M. (2017). Uncertain Rule-Based Fuzzy Systems: Introduction and New Directions.

[32] Karnik N.N., Mendel J.M., Liang Q. (1999). Type-2 fuzzy logic systems. IEEE Trans. Fuzzy Syst..

[33] Mendel J.M., John R.I., Liu F. (2006). Interval type-2 fuzzy logic systems made simple. IEEE Trans. Fuzzy Syst..

[34] Kumbasar T., Hagras H. (2014). A self-tuning zSlices-based general type-2 fuzzy PI controller. IEEE Trans. Fuzzy Syst..

[35] Wu D. (2012). On the fundamental differences between interval type-2 and type-1 fuzzy logic controllers. IEEE Trans. Fuzzy Syst..

[36] Noshadi A., Shi J., Lee W.S., Shi P., Kalam A. (2014). Genetic algorithm-based system identification of active magnetic bearing system: A frequency-domain approach. Proceedings of the 11th IEEE International Conference on Control & Automation (ICCA).

[37] Eberhart R., Kennedy J. Particle swarm optimization. Proceedings of the IEEE International Conference on Neural Networks.

[38] Saremi S., Mirjalili S.Z., Mirjalili S.M. (2015). Evolutionary population dynamics and grey wolf optimizer. Neural Comput. Appl..

[39] Atashpaz-Gargari E., Lucas C. (2007). Imperialist competitive algorithm: An algorithm for optimization inspired by imperialistic competition. Proceedings of the 2007 IEEE Congress on Evolutionary Computation.

[40] Yu Y., Xu Z.B., Wu Q.W., Yu P., He S., Wang G.Q. (2017). Kinematic analysis and testing of a 6-RR RP RR parallel manipulator. Proc. Inst. Mech. Eng. Part C J. Mech. Eng. Sci..

[41] Wang Q., Yu H., Wang M., Qi X. (2019). An improved sliding mode control using disturbance torque observer for permanent magnet synchronous motor. IEEE Access.

[42] Wen S., Zhang B., Hao P., Lam H.K., Wang H. (2017). Fuzzy fractional order force control of 6PUS-UPU redundantly actuated parallel robot based on inner model position control structure. Eng. Appl. Artif. Intell..

[43] Jan R.M., Tseng C.S., Liu R.J. (2008). Robust PID control design for permanent magnet synchronous motor: A genetic approach. Electr. Power Syst. Res..

[44] Wen S., Qin G., Zhang B., Lam H.K., Zhao Y., Wang H. (2016). The study of model predictive control algorithm based on the force/position control scheme of the 5-DOF redundant actuation parallel robot. Robot. Auton. Syst..

